# Eadyn’s Beacon: Illuminating Vasopressor management strategies

**DOI:** 10.1186/s13613-024-01410-2

**Published:** 2024-11-24

**Authors:** Rogério da Hora Passos, Sávio Sérgio Ferreira Custódio, Tomas de Azevedo Rodrigues, Igor Dovorake Lourenço, Bruno de Arruda Bravim, Thiago Domingos Correa

**Affiliations:** Hospital Israelita Albert Einstein, São Paulo, Brazil

**Letter to the Editor:**

Dear Editor,

I commended Alvarado-Sánchez et al. for their insightful systematic review on the predictive utility of Eadyn for vasopressor withdrawal in critically ill patients. Their work *provided valuable preliminary data and contributed* significantly to our understanding of hemodynamic management in intensive care settings. However, *several key areas merited further consideration to enhance the impact* and applicability of their findings.

*The review’s* inclusion of *only* five studies with a total of 183 patients *offered initial insights but may have limited the generalizability of the conclusions*. Expanding the inclusion criteria *to encompass a broader range of studies could have reduced* selection bias and *improved* the robustness *of the findings*. Additionally, the predominant focus on septic shock patients (76%) *narrowed the applicability of Eadyn’s predictive utility to other shock states. Future research should explore its effectiveness* in hypovolemic and cardiogenic shock *to fully assess its generalizability* [1, 2].

*One notable concern was* the variation in measurement methods for Eadyn across studies. *This inconsistency introduced potential bias and affected the reliability of the results*. Standardizing measurement techniques *could have enhanced* the consistency and reproducibility of future research. *The review identified* a low level of heterogeneity (I² = 0%), but this *may have reflected* the small number of studies rather than true homogeneity. *Further investigation into potential sources of variability*, such as differences in patient populations and intervention protocols, *could have provided* a clearer understanding of the findings. *Additionally*,* the funnel plot suggested possible publication bias. Employing more sophisticated techniques*,* such as Egger’s regression test*,* could have provided a more nuanced evaluation of this potential bias*.

The promising predictive ability of Eadyn (AUC = 0.85) for vasopressor withdrawal *was noteworthy*. However, its practical application across varied ICU settings *required* further investigation. Variations in management practices, including fluid resuscitation and vasopressor weaning protocols, *may have impacted* Eadyn’s effectiveness. *Moreover*,* the review’s* exclusive focus on norepinephrine *left* unanswered questions about Eadyn’s predictive value with other vasopressors, such as vasopressin or epinephrine.

*The current focus on* mean arterial pressure (MAP) reduction during vasopressor weaning, while valuable, *provided an incomplete picture*. *A more comprehensive assessment should have included additional outcomes* like weaning duration, ICU length of stay, and patient survival to better understand Eadyn’s impact on patient care [2, 3].

*Although* Eadyn *offered* valuable insights into arterial load and ventricular-arterial coupling, it was not without limitations. *Factors such as heart rate variability and arterial stiffness could affect its reliability*. The interplay between Eadyn and fluid responsiveness *was* complex; *therefore*,* future research should have integrated fluid responsiveness assessments to improve the accuracy and applicability of Eadyn in predicting* vasopressor withdrawal [4, 5].

*In summary*, while Alvarado-Sánchez et al.’s study *provided important* preliminary insights, *addressing the methodological concerns and exploring Eadyn’s applicability across different clinical settings and vasopressor types will be crucial* for establishing it as a standard tool for vasopressor *weaning*. I appreciated the authors’ contributions to this critical area of research and looked forward to further studies that built on these findings.

Sincerely,

## Data Availability

Not applicable.

## Abbreviations

Eadyn: Dynamic arterial elastance
AUC: Area under the curve
MAP: Mean arterial pressure
ICU: Intensive care unit
